# Relations between the levels of moderate to vigorous physical activity, BMI, dietary habits, cognitive functions and attention problems in 8 to 9 years old pupils: network analysis (PACH Study)

**DOI:** 10.1186/s12889-024-18055-2

**Published:** 2024-02-21

**Authors:** Jelena Raudeniece, Edmunds Vanags, Ilze Justamente, Dana Skara, Per Morten Fredriksen, Iain Brownlee, Dace Reihmane

**Affiliations:** 1 Department of Human and Animal Physiology, Faculty of Biology, University of Latvia, Jelgavas Street 1, Riga, LV-1004 Latvia; 2 Department of Human Physiology and Biochemistry, Riga Stradiņš University, Dzirciema Street 16, Riga, LV-1007 Latvia; 3 Department of Psychology, Faculty of Education, Psychology and Art, University of Latvia, Jelgavas Street 1, Riga, LV-1004 Latvia; 4 Department of Biotechology, Faculty of Applied Ecology, Agricultural Sciences and Biotechnology, Inland Norway University of Applied Sciences, 2318 Hamar, Norway; 5 Department of Applied Sciences, Northumbria University, Newcastle upon Tyne, NE1 8ST UK

**Keywords:** Emotional health, Cognitive functions, Structured physical activity, Dietary habits, Body mass index, Pupils, Elementary school

## Abstract

**Background:**

Physical activity (PA) and dietary habits (DH) play a crucial role on quality of life and health outcomes from various aspects.

**Methods:**

This study aims to investigate the relations between recommended daily levels of moderate to vigorous physical activity (MVPA) in 8 to 9 year old pupils, and their body mass index (BMI), DH, cognitive functions and attention problem scores by network analysis. Study participants were split into two groups based on their MVPA levels on weekdays.

**Results:**

Our findings suggest that children who reach recommended MVPA levels consume more vegetables and fruits, eat breakfast more frequently, have better motor speed and lower impulsivity score.

**Conclusions:**

The number of interlinkages between various parameters in network structure for children who do not reach recommended MVPA levels is greater and more intense, highlighting the differences between the groups and suggesting that different interventions and approaches to improve/change lifestyle habits might be used.

**Supplementary Information:**

The online version contains supplementary material available at 10.1186/s12889-024-18055-2.

## Background

PA and DH are considered as the main contributors to children’s physical and mental development, and cognitive and learning skills [1, 2]. PA and DH interact with each other and have to be balanced to form positive behaviour [3]. Childhood is a predominant and sensitive period of formation of lifestyle habits that lasts into adulthood, thus it is important to form prudent lifestyle habits in children [4, 5]. Furthermore, regular PA and prudent food choices decrease risk of non-communicable diseases and increase the possibility to live a more qualitative life throughout the life cycle [6, 7].

We live in an age of technological development where children more than ever are exposed to an inactive lifestyle due to new technologies and easily accessible inactive social life through internet [8]. Decreased PA increases the risk of several health issues, e.g., overweight and obesity, mental health problems and posture defects such as scoliosis. Moreover, physically inactive children who are overweight are more prone to have harmful behaviours, such as attempting suicide and developing addictions to alcohol and drugs [9].

Successful mental and emotional maturation requires individuals to develop cognitive processes and executive functions as motivation, ability to set goals, self-control, flexible thinking, time management, and organisation skills from early childhood while neural networks of executive functions are still developing [10, 11]. Appropriate PA maintains cognitive health throughout the life cycle and decreases risk of mental diseases [12]. Previous studies indicate that organised PA has a positive effect on executive functions, speech, skill to learn languages and in general improves academic performance [13]. Contrarily, a lack of PA in childhood is associated with limited perception and development disorders [9, 14]. Along with physical activities, DH play no less significant role. Prudent habitual dietary choice, characterised by alignment with food-based recommendations and consumption of nutrient-dense food promotes adequate mental and physical development in a period of rapid growth in children [15].

Studies have indicated an inverse association between increased PA and attention problems and academic performance [16], as well improved cognitive and behavioural outcomes in children with attention deficit hyperactivity disorder (ADHD) [2, 17, 18]. Imprudent DH, such as high sugar and saturated fat intake, has been linked to an increased risk of ADHD, while, for example high fruit and vegetable consumption, may reduce the risk of such issues [19, 20]. Although ADHD is a medical diagnosis and is diagnosed by complex questionaries and interviews by clinicians, attention problems are considered as one of the ADHD symptoms [21].

Network analysis and computational models have gained popularity in contemporary intervention studies. These models help to understand the complex relationship between social influences and various factors that impact certain health behaviours [22]. These methods allow researchers to collect initial data, simulate different interventions, and choose the most effective or cost-efficient strategies to change behaviour. The current study aimed to evaluate an existing cross-sectional dataset for network relationships between recommended daily levels of moderate to vigorous physical activity in children (8-9y), their body weight status, dietary habit, cognitive abilities and attention problems.

## Materials and methods

### Study design and participants

Cross-sectional quantitative data (baseline characteristics of pupils participating in the Physical Activity and Children Overall Health (PACH study) were used for this network analysis. Convenience sampling method was used to recruit 1563 pupils (8 to 9 years old) from 59 schools across Latvia. Among the participants, 53% were male (*n* = 826) and 47% were female (*n* = 737). Pupils participating in the PACH study were enrolled in the "Sport for All Class" (SFAC) project, which ensures 5 school-based physical education classes per week compared to the standard of 2 to 3 classes per week. To participate in the SFAC, schools had to submit an application with the list of participating pupils and corresponding signatures from their parents.

The PACH study is driven by a collaboration project between the University of Latvia and the Latvian Olympic Committee (ZD2019/20861) that adheres to ethical principles for medical research involving human participants, in line with the Declaration of Helsinki 2016. The Ethical Committee of the Institute of Cardiology and Regenerative Medicine, University of Latvia approved the PACH study (Nr.179/2019; effective from 14.10.2019). Parents were required to provide written agreement which included an explanation on all procedures and possible risks, while verbal agreement was obtained from the participants. Participants had the right to withdraw from the study at any point without explanation.

### Measures

#### Anthropometric measurements and physical activity levels

To determine (BMI, kg/m^2^) data on weight and height were collected and percentile was calculated according to World Health Organization (WHO) growth reference data for 5–19 year-old children [23].

Levels of structured PA on weekdays were determined by a questionnaire. Pupils' responses were used to calculate youth metabolic equivalents (METy) which represent cost of PA [24]. Additionally, the study estimated the prevalence of pupils who met the WHO's recommendations for moderate to vigorous physical activity (MVPA) on weekdays. To investigate how the network model of the study variables differed depending on physical activity levels in children, the next stage of data analysis was performed in two sample groups. OPTIMAL-MVPA (*n* = 774) represents children who achieved the WHO's 5-day recommendation for MVPA on weekdays and LOW-MVPA (*n* = 789) represents children who did not. More detailed description of the survey items, answers and scores on METy has been published previously [25].

#### Dietary habits

Such DH as eating breakfast, fruit and vegetable consumption, choice of snacks and beverages, and eating culture (eating at the table alone or with family vs eating while watching TV or using mobile phone) were assessed by questionnaire. More detailed description of the survey items, answers, and adapted scoring system have been published previously [25].

#### Attention level

To determine attention problems the subscale Attention Problems of the Youth Self Report (YSR), (Achenbach, 1991; adapted in Latvia by Sebre and Laizane, 2006) was used. It consists of 9 statements with 3 possible answers – 0 = not true, 1 = somewhat or sometimes true, or 2 = very true or often true (Table A1). Accordingly, the minimum possible score was 0 and the maximum 18. Higher scores on this scale indicate attention problems. Although the recommended age for completing this questionnaire is 11–18 years old, some studies have already used this questionnaire for younger children without systematically assessed validation [26, 27]. Previous research has shown that Attention Problem subscale is a good tool to predict ADHD if reported by parents, but not by youth themselves [28]. The Cronbach's alpha reliability coefficient in this study was α = 0.76, which allows its use for further data processing.

#### Cognitive functions

All cognitive functions were assessed using computerised versions of tests for psychological testing provided by SIA Exploro (exploro.lv). Participants were familiarised with the procedure of each cognitive test by filling out the sample test to ensure compliance and comparability across repeats.

##### Motor speed and motor control

The Finger Tapping test is a simple neuropsychological measure of self-directed manual motor speed and motor control [29].This test was used as a sensitive measuring tool in case of brain damage. During the test, the participants were asked to tap the keyboard as many times as possible with an index finger for 10-s intervals. The test must be repeated 3 consecutive times with each hand. There was a 3-s pause between each attempt to rest the hand. The results were expressed as the number of taps for each stage.

##### Selective attention and cognitive flexibility

The measure of selective attention and cognitive flexibility was first developed in 1935 by Stroop [29]. Many versions of the Stroop test are currently available. The computerised test used in this study consisted of 3 stages all presenting only the names of various colours. In the 1st stage all colour names were black, and one must press TAB on the keyboard, as soon as any word appears on the screen. Within this stage, the response time of the reaction to stimulus was measured. In the 2nd stage, the participants are asked to press TAB on the keyboard only when the name of the colour matched with the colour of the writing (e.g., the word blue was coloured blue and not yellow), while if in case the name of the colour did not match the colour it is written with (e.g., the word blue was in red colour), the stimulus should be ignored. The measurements obtained in the 2nd stage were the number of the correct and incorrect responses and the response time. In the 3rd stage, the participants were instructed to press a TAB on the keyboard only when the name of the colour was written in the wrong colour and to ignore the correct match. In the 3rd stage information on processing speed, inhibition control, selective attention, and executive functions in general was collected. This test is also considered as a specific and reliable method for differentiating children with ADHD from children without ADHD [30].

##### Information processing speed

The Symbol Digit Modalities test was originally designed to detect cerebral dysfunction in children and adults. This test involves perceptual and processing speed, motor speed, and working memory, as well as visual scanning and tracking. At the top of the screen a grid of 9 symbols (top row) and corresponding 9 numbers (bottom row) was displayed throughout the test. When a symbol appeared at the bottom of the screen, participants were asked to enter the corresponding digit of the symbol in the blank box as fast as possible (Figure A1), with the number of correct and incorrect digits in 2 min measured alongside input times (in milliseconds).

### Procedure

Pupils were examined once in a school environment, during the visit of the research team from November 2019 to January 2020. Initially, children completed cognitive tests online using exploro.lv. Following a break, they proceeded to answer electronic questionnaires that estimated their DH, PA levels, and symptoms of attention problems. The researchers and class teachers were present to assist the pupils in completing the tests and questionnaires. Lastly, the study research team provided instructions to the medical nurses working in the schools, who participated in gathering anthropometric measurements according to a standardised protocol (COSI) [31] using equipment available in the local schools.

### Statistical analysis

The JASP version (v. 0.17.1, University of Amsterdam, Amsterdam, The Netherlands) on macOS Big Sur version (v. 11.5.2, Apple Inc., Cupertino, California, United States) was used for data analysis [32]. For determination of data distribution, the Shapiro–Wilk test was used. Respectively, p -value with significance level < 0,05 represents non normal distribution of data. Furthermore, means, standard deviations for variables, internal consistency for scales, Spearman correlations, and confidence intervals were determined. Mann–Whitney U test was performed to indicate a significant difference between OPTIMAL-MVPA and LOW-MVPA groups.

#### Network analysis

To identify the relationships from the mutualism perspective between variables included in the study network structure analysis using high-dimensional undirected graph estimation was performed using the glasso (or graphical lasso) procedure that estimates a network in which the edges are partial correlation coefficients [33]. Undirected network analysis is a commonly used approach to describe the conditional independence and interrelationships of many variables. The graphical representation of networks is based on the Fruchterman – Reingold algorithm [34] that places nodes with stronger and/or more connections closer together. Each node in the graph represents one variable (or symptom) included in the analysis. Each edge represents the relationship between two variables, controlling for all other relationships in the network and no connection between two variables means that they are conditionally independent of all other variables. The role of each node or symptom in the network structure is determined using indicators of centrality [34]:

- Strength—How strongly a node was directly connected (absolute sum of edge weights connected to a node);
- Closeness—How strongly a node was indirectly connected (average distance from the node to all other nodes in the network);
- Betweenness—How well one node connected other nodes (the number of times that a node lay on the shortest path between two other nodes);
- Expected influence—the sum of all edges extending from a given node (maintaining the sign). Expected influence computed node strength without taking the absolute value of edge-weights.

The tuning parameter 0,1 was chosen using the Extended Bayesian Information Criteria (EBIC) [34]. The concept behind the network model is that nodes have important connections with one another, continuously interact, and these dynamic relationships result in observable behaviour. By adopting this model, we could gain a better understanding of the possible behaviour patterns from a mutualistic viewpoint.

The network analysis was based on a partial correlations’ matrix (Figure A2). The network graph can depict not only indirect relationships, but also mediation pathways, thus serving as a valuable tool to generate hypotheses regarding the potential causal structure of specific nodes [34, 35]. The various nodes in a network may play distinct roles, and measures of centrality can provide an insight into the relative significance of these nodes [36].

#### Reliability and validity

To assess the accuracy and stability of a particular network structure analysis with bootstrapping was used, allowing to estimate the model under simulated and sampled data. The sampling distribution of the particular parameters was estimated performing the nonparametric bootstrapping with 1000 samples. Stability check of the centrality indices by dropping cases from the dataset showed that strength and closeness indices were relatively stable as correlation did not drop below 0.7 when the subset sample decreased by 50% from the original sample. In turn, betweenness indices were less stable, as correlation dropped below 0.7 when the subset sample decreased by 30% from the original sample. In general, strength and closeness estimates decrease at a lower rate even when 25% of the original sample is used, but betweenness estimates decrease at a higher rate and greater number of original samples. In psychology networks, the centrality index of strength is typically estimated with high precision, while the estimation of betweenness and closeness only becomes reliable in larger samples [34] (Figure A3). Bootstrapped difference tests (with α = 0.05) between the edge weights and centrality measures in the estimated network showed notable differences in the node strength particularly for processing speed error, executive function, and processing speed nodes (Figure A4).

## Results

### Descriptive statistics

According to the Shapiro—Wilk test (*p* < 0.05), data significantly deviated from normal distribution. Significant differences between OPTIMAL-MVPA and LOW-MVPA in multiple parameters were observed (Table 1).

**Table 1:** Descriptive statistics of OPTIMAL-MVPA and LOW-MVPA

Parameter	Median (25th; 75th quartile)		Mean (min; max value)		Mann–Whitney
	**OPTIMAL-MVPA**	**LOW-MVPA**	**OPTIMAL-MVPA**	**LOW-MVPA**	***p* value**
BMI, kg/m^2^	16.4 (15.3; 18.1)	16.5 (15.1; 18.4)	17.0 (12.1; 31.4)	17.2 (11.9; 31.9)	0.783
Light activities, min	295.0 (95.0; 310.0)	250.0 (250.0; 280.0)	211.1 (20.0; 310.0)	260.2 (20.0; 310.0)	< 0.001
Moderate activities, min	375.0 (330.0; 430.0)	175.0 (150.0; 250.0)	384.5 (140.0; 695.0)	197.1 (60.0; 295.0)	< 0.001
Vigorous activities, min	0.0 (0.0; 0.0)	0.0(0.0; 0.0)	32.1 (0.0; 160.0)	8.8 (0.0; 120.0)	< 0.001
Frequency of breakfast^a^	3.0 (2.0; 3.0)	3.0 (1.0; 3.0)	2.4 (0.0;3.0)	2.3 (0.0; 3.0)	0.004
Consumption of vegetables, portions/d	2.0 (1.0; 3.0)	2.0 (1.0; 3.0)	2.2 (0.0; 4.0)	2.0 (0.0; 4.0)	0.003
Consumption of fruits, portions/d	2.0 (2.0; 3.0)	2.0 (1.0; 3.0)	2.2 (0.0; 3.0)	2.0 (0.0; 3.0)	< 0.001
Choice of positive snacks, number^b^	1.0 (1.0; 2.0)	1.0 (1.0; 1.0)	1.1 (0.0; 3.0)	1.0 (0.0; 3.0)	0.855
Choice of negative snacks, number^c^	1.0 (0.0; 1.0)	1.0 (0.0; 1.0)	0.8 (0.0; 3.0)	0.8 (0.0; 3.0)	0.459
Choice of sugar drinks^d^	1.0 (0.0; 2.0)	1.0 (0.0; 2.0)	1.0 (0.0; 2.0)	1.0 (0.0; 2.0)	0.423
Eating in front of the screen^e^	1.0 (1.0; 2.0)	1.0 (1.0; 2.0)	1.3 (1.0; 2.0)	1.3 (1.0; 2.0)	0.170
Attention level	7.0 (4.0; 9.0)	7.0 (4.0; 9.0)	6.6 (0.0; 18.0)	6.8 (0.0; 18.0)	0.135
Motor speed, taps/10 s	50.2 (46.2; 54.2)	48.7 (44.8; 52.8)	49.5 (0.2; 72.2)	48.5 (0.0; 73.2)	< 0.001
Processing speed, correct symbols/2 min	40.0 (35.0; 45.0)	39.0 (34.0; 44.0)	39.8 (0.0; 68.0)	39.4 (0.0; 77.0)	0.210
Processing speed errors, incorrect symbols/2 min	2.0 (0.0; 3.0)	1.0 (0.0; 3.0)	2.9 (0.0; 108.0)	1.8 (0.0; 19.0)	0.080
Executive functions, total correct responses	23.0 (21.5; 23.5)	22.5 (21.5; 23.5)	21.9 (0.0; 24.0)	21.7 (1.0; 24.0)	0.334
Impulsivity, total incorrect responses	1.0 (0.0; 3.0)	1.0 (0.0; 3.0)	5.2 (0.0; 166.0)	5.8 (0.0; 181.0)	0.031

^a^1—never; 2—sometimes; 3—almost every day; 4—every day

^b^sum of nuts/dried fruits, fruits, yoghurt

^c^sum of cookies, sweet bakery, salty nuts, French fries, popcorn, chips, chocolate, chocolate sweets, ice—cream

^d^0—water; 1—tea; 2—soft drinks/sweetened juices

^e^1—no, 2—yes

Children from LOW-MVPA spend significantly less time in light, moderate and vigorous physical activity, while children from OPTIMAL-MVPA spend more time in all previously mentioned types of physical activity and consume more vegetables, fruits daily and eat breakfast more frequently. Average BMI and attention problem score is similar for both groups. The average motor speed is significantly better for the children from OPTIMAL-MVPA compared to LOW-MVPA. Although the children of both groups have managed to process almost the same amount of information on average, the children from LOW-MVPA tended to make fewer errors while processing this information. The average score of executive functions of both groups was similar, however OPTIMAL-MVPA showed significantly lower impulsivity score (Table 1).

Accordingly, to network analysis, 17 nodes formed 106 significant connections (out of 136 possible connections) with 0.22 sparsity. Partial correlations reflect the intensity of connections between variables while considering the influence of other measured variables in the network model. Consequently, nodes in the graph were linked only if there exists a correlation between them that cannot be accounted for any other variable in the network. Positive partial correlations were indicated as blue lines, while negative ones were indicated as red lines. The thickness of the lines reflects the degree of partial correlation, with thicker lines indicating stronger relations and thinner lines indicating weaker ones.

### Network analysis in whole sample

**Fig. 1:**
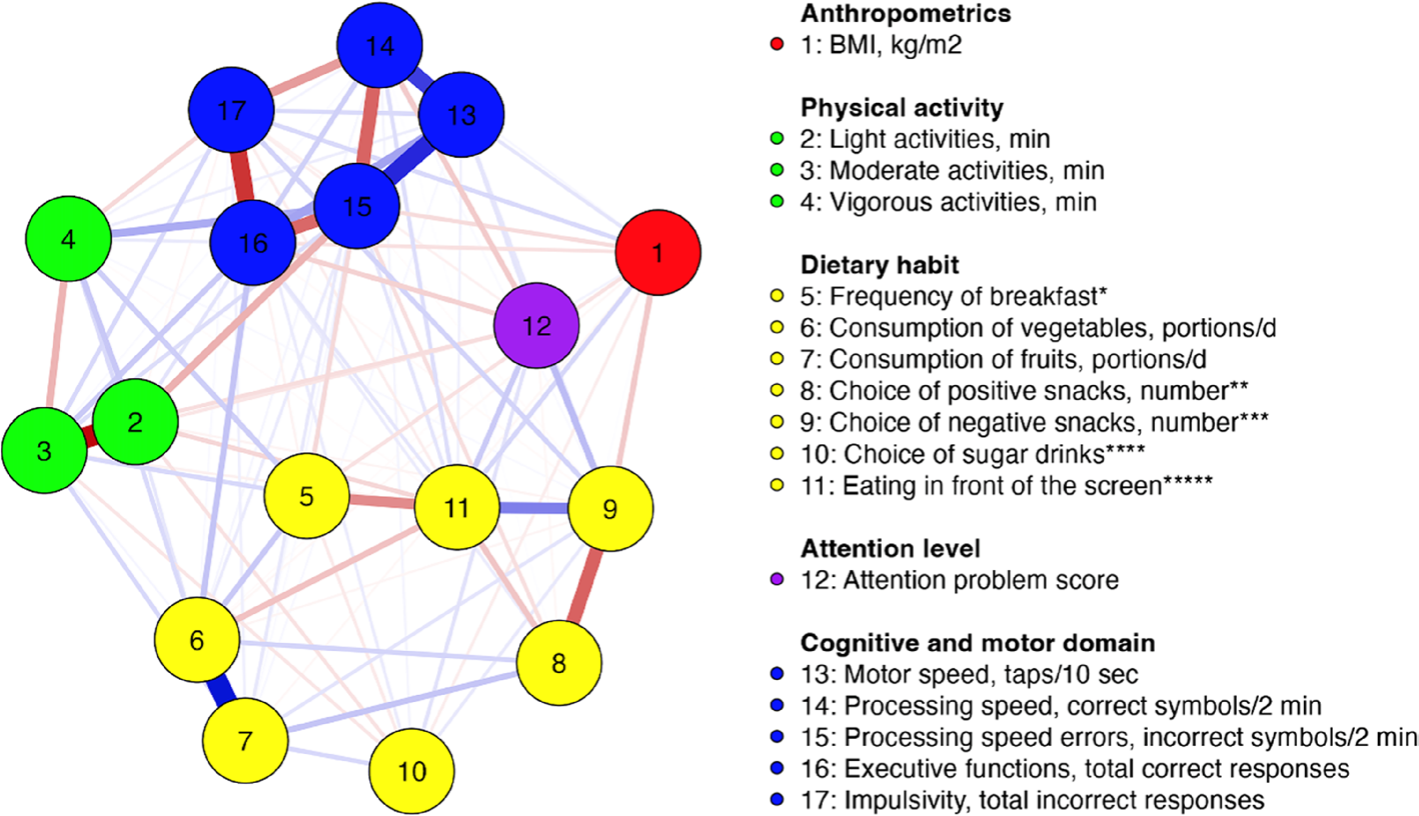
Network graph of study measures in the whole sample. Positive partial correlations are indicated as blue lines, while negative ones are indicated as red lines. The thickness of the lines reflects the degree of partial correlation, with thicker lines indicating stronger relations and thinner lines indicating weaker ones. * 1 - never; 2 - sometimes; 3 - almost every day; 4 - every day. ** sum of nuts/dried fruits, fruits, yoghurt. *** sum of cookies, sweet bakery, salty nuts, French fries, popcorn, chips, chocolate, chocolate sweets, ice – cream. **** 0 - water; 1 - tea; 2 - soft drinks/sweetened juices. ***** 1 - no, 2 - yes

Figure 1 represents significant partial correlations, relations between variables which have an impact on the rest of the variables. Variables linked by the same domain form closer correlations. For instance, close negative correlation was observed between light and moderate physical activities (-0.430), the edge between these nodes is not even visible in the network graph as nodes are located very close to each other, so data on correlation can be seen in Table 2. In turn, a positive correlation was observed between the motor and processing speed (0.327). A close partial correlation was between vegetable and fruit consumption (0.426). Nodes which represent consumption of positive and negative snacks had negative correlation (-0.265). Node of negative snacks was positively associated with attention problem score (0.112). Whereas, eating in front of the screen was negatively related to eating breakfast (-0.194), but positively related to consumption of negative snacks (0.216).

**Table 2:** Centrality measures of variables in network structure for the whole sample

**Variable**				**Network**
	**Betweenness**^a^	**Closeness**^b^	**Strength**^c^	**Expected influence**^d^
BMI, kg/m^2^	-0.96	-1.61	-1.27	-0.37
Light activities, min	0.65	0.25	0.17	-1.52
Moderate activities, min	-0.29	-0.06	0.42	-0.84
Vigorous activities, min	-0.96	-0.15	-0.78	0.48
Frequency of breakfast^e^	-0.56	-0.12	-0.87	-0.34
Consumption of vegetables, portions/d	0.65	0.41	0.34	1.49
Consumption of fruits, portions/d	0.24	0.08	-0.31	1.63
Choice of positive snacks, number^f^	-0.56	-0.56	-0.87	-0.65
Choice of negative snacks, number^g^	0.51	0.09	0.16	0.15
Choice of sugar drinks^h^	-0.96	-2.76	-1.82	-0.06
Eating in front of the screen^i^	0.51	0.34	0.51	-0.12
Attention level	-0.96	-0.34	-0.97	-0.15
Motor speed, taps/10 s	-0.96	0.44	0.66	2.35
Processing speed, correct symbols/2 min	-0.16	0.43	0.82	0.06
Processing speed errors, incorrect symbols/2 min	2.79	1.53	2.05	-0.63
Executive functions, total correct responses	1.18	1.32	1.36	-0.51
Impulsivity, total incorrect responses	-0.16	0.71	0.39	-0.96

^a^Betweenness—How well one node connects other nodes (the number of times that a node lies on the shortest path between two other nodes)

^b^Closeness—How strongly a node is indirectly connected (average distance from the node to all other nodes in the network)

^**c**^Strength—How strongly a node is directly connected (absolute sum of edge weights connected to a node)

^d^Expected influence—the sum of all edges extending from a given node (maintaining the sign). Expected influence computes node strength without taking the absolute value of edge-weights

^e^1—never; 2—sometimes; 3—almost every day; 4—every day

^f^sum of nuts/dried fruits, fruits, yoghurt

^g^sum of cookies, sweet bakery, salty nuts, French fries, popcorn, chips, chocolate, chocolate sweets, ice—cream

^h^0—water; 1—tea; 2—soft drinks/sweetened juices

^i^1—no, 2—yes

By analysis of centrality measures in network structure, in this pattern the highest expected influence (expected influence considers the positive and negative edges) was observed in motor speed, consumption of fruits and consumption of vegetables (Table 2).

It is significant to note that the processing speed error node has the highest betweenness score, which suggests that this variable possibly is the most important node connecting other nodes in the entire model. The betweenness score is relatively high for executive functions, consumption of vegetables and light physical activities, followed by eating in front of the screen. Accordingly, there are variables that at the same time can positively and negatively affect the relationships of the other variables of this model.

Network graph showed that consumption of vegetables and fruits is significantly related to eating breakfast and consumption of positive snacks, which generally corresponds to prudent DH. The cluster of these variables showed negative correlation with eating in front of the screen, consequently representing a positive relation with such a node as consumption of negative snacks which in turn is positively related with consumption of sugar containing beverages. Furthermore, a relation was observed between clusters of imprudent DH and attention problem scores. Respectively, the more pronounced imprudent DH are, the more pronounced signs of attention problems.

### Network analysis in subsamples

Network graph and centrality table represent that there is no statistically significant difference between the two groups, but there are some noticeable nuances (differences). When compared to OPTIMAL-MVPA, the network structure in LOW-MVPA is denser and there are more intense connections between nodes, which means that variables in this subsample can influence each other more quickly and significantly. Different dynamics were observed within groups, for example, in OPTIMAL-MVPA BMI has a single weak relationship with eating in front of the screen (0.013), in turn in LOW-MVPA BMI is connected to several variables like eating in front of the screen (0.057), consumption of unhealthy snacks (-0.13), vegetables (-0.022), sweetened beverages (-0.14) and frequency of breakfast (-0.048). In the LOW-MVPA executive function and impulsivity nodes are forming more significant connections with other nodes, indicating their importance in that model.

Network analysis of LOW-MVPA shows strong interlinkages and sensitivity between all four clusters—anthropometrics, nutrition, attention level and cognitive and motor domain meaning that each cluster has a significant importance and influence on the specific nodes of other clusters.

**Fig. 2:**
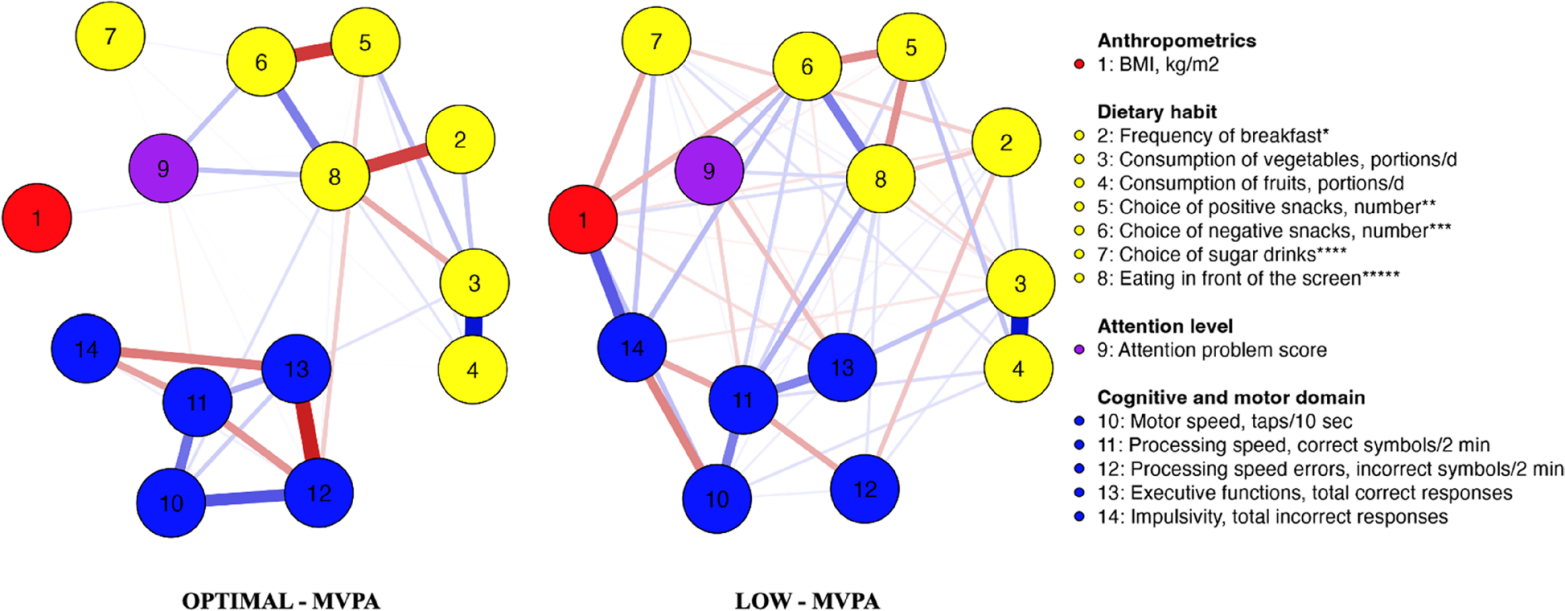
Network graph of study measures in OPTIMAL-MVPA and LOW-MVPA. OPTIMAL-MVPA—children who reach WHO recommendations for MVPA. LOW-MVPA—children who do not reach WHO recommendations for MVPA. Positive partial correlations are indicated as blue lines, while negative ones are indicated as red lines. The thickness of the lines reflects the degree of partial correlation, with thicker lines indicating stronger relations and thinner lines indicating weaker ones. * 1—never; 2—sometimes; 3—almost every day; 4—every day. ** sum of nuts/dried fruits, fruits, yoghurt. *** sum of cookies, sweet bakery, salty nuts, French fries, popcorn, chips, chocolate, chocolate sweets, ice – cream. **** 0—water; 1—tea; 2—soft drinks/sweetened juices. ***** 1—no, 2—yes

In LOW-MVPA BMI has a negative correlation with frequency of breakfast, consumption of vegetables, choice of negative snacks, and executive functions while positive correlation with impulsivity node. Such interlinkages cannot be observed in OPTIMAL-MVPA. Furthermore, positive correlation between BMI and eating in front of the screen is stronger in LOW-MVPA when compared to OPTIMAL-MVPA. Interpretation of linkages between BMI and other parameters might differ. For example, the less frequent breakfast, the less vegetables are consumed daily, the more children eat in front of the screen, the higher the BMI is and, accordingly, the executive functions get worse (deteriorate), but impulsivity increases. Furthermore, there is an interlinkage between eating breakfast less frequently and longer processing speed and increased number of processing speed errors in LOW-MVPA while such a relationship was not observed in OPTIMAL-MVPA (Fig. 2). This study found an unusual inverse relationship between BMI and choice of unhealthy snacks, and sugary drinks.

**Fig. 3:**
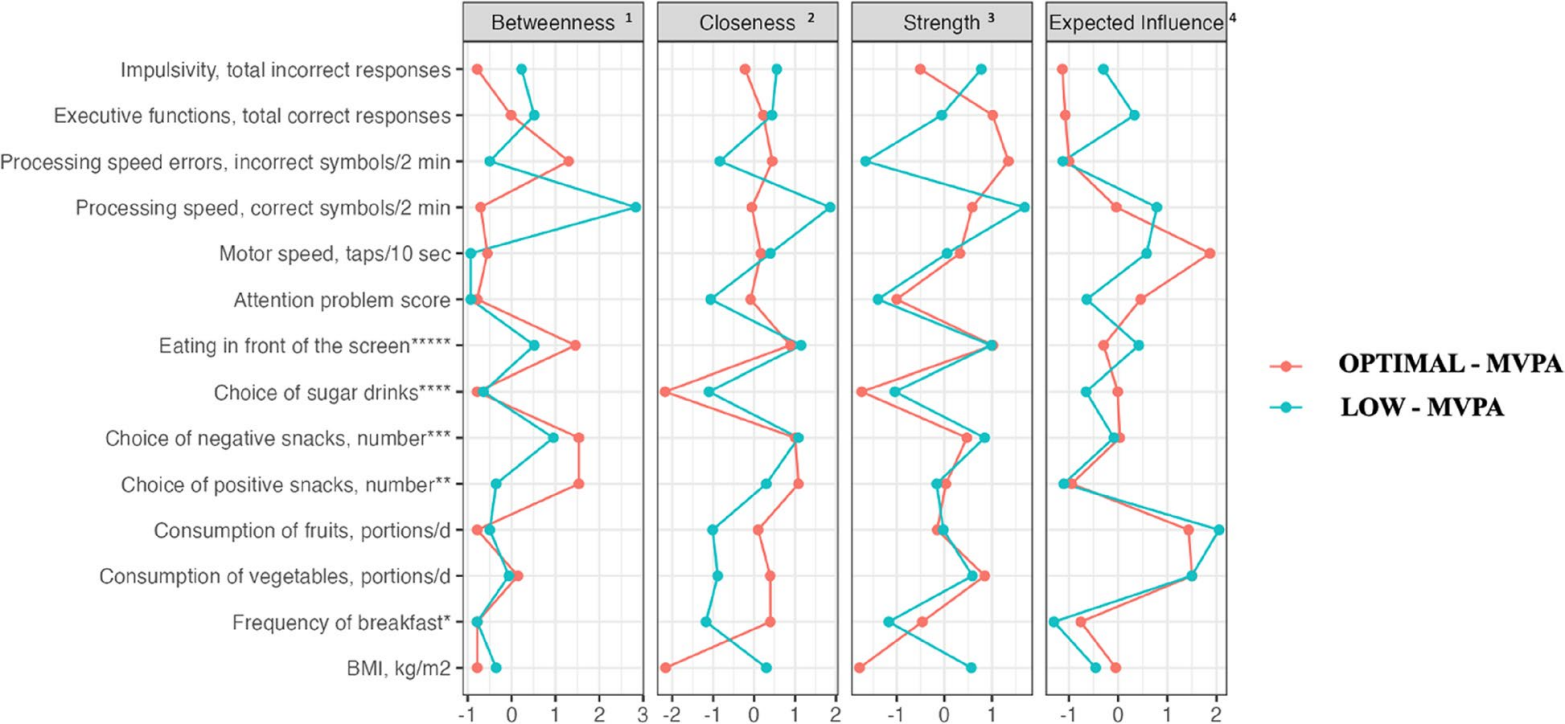
Centrality measures of variables in network structure comparing groups. ^1^ Betweenness—How well one node connects other nodes (the number of times that a node lies on the shortest path between two other nodes). ^2^ Closeness—How strongly a node is indirectly connected (average distance from the node to all other nodes in the network). ^3^ Strength—How strongly a node is directly connected (absolute sum of edge weights connected to a node). ^4^ Expected influence—the sum of all edges extending from a given node (maintaining the sign). Expected influence computes node strength without taking the absolute value of edge-weights. * 1—never; 2—sometimes; 3—almost every day; 4—every day. ** sum of nuts/dried fruits, fruits, yoghurt. *** sum of cookies, sweet bakery, salty nuts, French fries, popcorn, chips, chocolate, chocolate sweets, ice – cream. **** 0—water; 1—tea; 2—soft drinks/sweetened juices. ***** 1—no, 2—yes

Network graph of LOW-MVPA showed positive correlation between eating in front of the screen and executive functions, such correlation was not observed in OPTIMAL-MVPA. Another difference between network graphs of OPTIMAL-MVPA and 2 are betweenness scores representing nodes which connect other variables in the network model (Fig. 3). The node with the highest betweenness score in LOW-MVPA was for processing speed, but in OPTIMAL-MVPA for choice of positive and negative snacks, followed by eating in front of the screen.

Despite the marked differences shown by network analysis, similarities between the two groups can also be seen. Negative correlation can be observed between eating in front of the screen and such parameters as the frequency of breakfast and consumption of vegetables and positive snacks, suggesting that spending more time in front of the screen results in less prudent DH. In addition, eating in front of the screen is related to increased consumption of negative snacks and greater attention problem scores in both groups.

Nodes with one of the highest expected influence scores, in all provided network graphs (Table 2, Figs 1 and 3) are consumption of vegetables and fruits, indicating that these DH play a central role in a particular model and these two nodes have a power to impact other nodes in this model, despite the marked differences in the number of interlinkages formed with other parameters.

## Discussion

The tandem of PA and prudent DH have a positive effect on cognitive functions and academic performance, numerous studies showed that MVPA promotes more prudent eating choices like higher consumption of fruits, vegetables, grains, fish, dairy products and water, on other hand low PA and sedentary lifestyle is associated with consumption of soft drinks, fast food, low nutrition and high energy snacks [3, 37, 38]. Such dietary choices have low nutritional value and are associated with decreased executive functions and poor academic performance [39]. Previous studies suggest that PA has positive effects on memory, attention, overall improved cognitive flexibility in and inhibitory control, in turn low PA levels are associated with deterioration of cognitive functions [1, 9]. The outcome of systematic review reports that regular MVPA contributes to improved cognitive ageing [12]. The present study aimed to explore the complex interplay between BMI, DH, cognitive functions and attention problem scores in 8–9-year-old children with different levels of MVPA.

### Network analysis in the whole sample

Variables with the highest expected influence score in the whole sample were motor speed, consumption of fruits and vegetables, suggesting that in such a pattern of activities, changing the pattern of motor speed and/or consumption of vegetables and fruits can affect and change relations between other nodes sequentially. In turn, light and vigorous PA time, choice of healthy snacks and impulsivity had the least expected influence, meaning that possibly these nodes were not central factors and would not be primary targets of intervention.

Cluster representing DH: consumption of vegetables, fruits, positive snacks, and frequent breakfast eating showed a negative relation with eating in front of the screen, which was positively associated with negative snacks, but this variable had positive correlation with consumption of sugar containing beverages. Moreover, cluster of imprudent DH and attention problem score showed positive interlinkage. These data can be used to think about two-way effects on causal relationships. For example, a higher attention problem score appears to be associated with negative snacking in front of the screen, which in turn might reduce the potential consumption of positive snacks, vegetables, and fruits or second scenario—eating in front of the screen is associated with consumption of unhealthy snacks which in turn promotes higher attention problem score.

As well, two-way effects can be considered in the relationship of light, vigorous physical activities and executive functions, accordingly higher levels of executive functions can promote children to get involved in PA or PA promote development of executive functions. Significantly to mark that this “bridge” is linked with higher levels of processing speed, lower levels of processing speed errors and lower impulsivity.

### Comparison of network graphs between OPTIMAL-MVPA and LOW-MVPA

#### Comparison of network graphs in respect to BMI

There are several factors impacting BMI. Some of them cannot be controlled like developmental determinants, genetic material, gender, and age, but PA and diet can and should be controlled. Over the years studies have reported that insufficient amounts of nutrients, tendency to skip breakfast, energy dense snacking as well as decreased PA levels [38, 40–43] and eating whilst watching TV [44] are positively associated with increased BMI in children. Despite our observations of interconnections between frequency of breakfast and decreased consumption of vegetables and fruits in children with insufficient MVPA levels, the BMI was not different between the two study groups. It might suggest that daily PA is one of the major health and behaviour determinants in children irrespective of BMI. Furthermore, mentioned differences can indicate that children who reach recommended MVPA levels would not experience an increase in BMI by temporary change of DH, which is in line with other studies that generally show that more physically active children have more prudent DH [25, 37]. Furthermore, both PA levels [16] and BMI [45] have been shown to be related to executive functions, in turn this node has relatively high betweenness score and serves as bridge between other nodes like consumption of vegetables and attention problem node, which in turn is consistent with the central role of executive function in decision making and control of behavioural processes [46]. Our study data revealed relatively high betweenness scores for executive function. In summary, previously described interconnections might suggest that children who are less physically active tend to skip breakfast more often, while more often choose to eat in front of the screen, resulting in higher BMI and impaired executive functions.

In addition previous studies have reported that doing sports promotes cognitive and emotional development, as well as increases cognitive performance, as children tend to be more motivated and self-controlled [9, 47, 48]. Nevertheless, BMI score is similar in both groups, BMI node is not linked with cognitive and motor domain in OPTIMAL-MVPA, representing “stability” of children who reach 300 min of recommended MVPA on weekdays.

Interestingly, BMI had an inverse relationship with the choice of negative snacks in children who do not reach the WHO recommendations for MVPA. Research from the 2000s in Norway and Great Britain found a similar phenomenon, when sugar consumption negatively correlated with BMI in adolescents [49]. A possible explanation for our finding could be that children with higher BMI could underreport on their actual daily choice of drink or children selected beverages which they would prefer to have and not the one they use daily.

#### Comparison of network graphs in respect to fruit and vegetable consumption

It has been shown that family lifestyle, nutritional knowledge, and parental intake of vegetables and fruits motivates children to consume more vegetables and fruits, suggesting that parents are role models to their children [50]. Furthermore, fruit and vegetable availability at home and shared family meals are associated with increased fruit and vegetable consumption in children and adolescents [51]. On the other hand if a family consumes less fruits and vegetables, such a habit can promote other imprudent eating habits, for example less frequent family meals, more eating in front of the screen and consumption of negative snacks [52] which is in line with our findings as nodes of vegetables and fruits have an impact on many other nodes.

Consumption of fruits and vegetables is also highly associated with PA levels. Our data support findings by a study in Germany which reported that children who reach recommended PA levels are more prone to consume even by 50% more fruits and vegetables [3]. Low fruit and vegetable consumption is associated with insufficient amount of total and insoluble fibre intake as well as with higher risk of obesity and related diseases. Low fibre intake in turn is associated with poor academic performance and cognitive functions [53, 54] which is in line with a positive association found in our study between eating fruits, vegetables and executive functions in both study groups. In turn, fruit and vegetable consumption is significantly lower and is positively associated with cognitive processing and motor speed in LOW-MVPA, but not in OPTIMAL-MVPA. Also, an inverse association between consumption of fruits was found with BMI node in LOW-MVPA, but not in OPTIMAL-MVPA. Accordingly, this possibly represents stability of OPTIMAL-MVPA and could mean that by reaching recommended MVPA, children have lower risk of increasing BMI, as well as becoming slower in motor skills and slower inability processing information, even if the daily consumption of vegetables and fruits gets reduced. Our findings support evidence that PA has a positive effect on cognitive functions, such as improved task completion time, increased ability to focus attention on specific tasks, and regular reaching of MVPA promotes physiological cognitive ageing [1, 9]. There is a limited amount of information available about the interaction of the two factors as PA and DH and their effect on cognitive functions, but in this study, after comparing OPTIMAL-MVPA and LOW-MVPA, it could be concluded that in children who reach recommended MVPA, when consumption of fruits and vegetables decrease, motor functions and information processing time will not be affected to such an extent.

#### Comparison of network graphs in respect to eating in front of the screen

Despite the finding that eating in front of the screen is similar in both groups, more physically active children would still make more prudent food choices. IDEFICS and KiGGS studies reported that physically active children are more into beneficial foods [3], but less physically active children have higher consumption of fast food [55] which is in line with our findings as negative association was observed between eating in front of the screen and consuming more positive snacks and vegetables in LOW-MVPA, but not in OPTIMAL-MVPA. And it should be emphasised that physical activities of a certain intensity, which are achieved on weekdays, play an important role here, which is the main difference between both study groups. If children who reach recommended MVPA will start eating in front of the screen they will be less exposed to the risk of consuming negative snacks, then children who do not reach recommended MVPA.

#### Comparison of network graphs in respect to frequency of breakfast

Unequivocally, both factors – PA and DH, are important for children’s lifelong health. Our data support findings by previous studies suggesting that more physically-active children eat breakfast more regularly and have better cognitive performance [44, 54, 56] as well as breakfast eating is associated with higher MVPA levels [57]. Although MVPA also may help with decreasing ADHD symptoms and improving processing speed in children with ADHD [58], our study did not find any differences in attention problem score and processing speed between the groups.

Comparison of network graphs between groups showed no interconnections between frequency of breakfast node and cognitive domain in OPTIMAL-MVPA. Furthermore, the processing speed error node is negatively associated with processing speed, which is positively related to executive functions in both groups. At the same time executive function node negatively correlates with attention problem node. Accordingly, the more often breakfast will be skipped, the more processing speed errors will be made by children in LOW-MVPA, the slower processing speed will become, the weaker executive functions will get, which potentially result in higher attention problem score. According to the data analysis methods chosen in this study, it might be suggested that children who met MVPA recommendations, with a short-term change in the regularity of breakfast eating, will not make more processing speed errors. Thus, in children who do not reach MVPA recommendation levels, irregularity of breakfast eating might have more significant impact on the processing speed errors and consequently more errors when processing information.

Breakfast skipping is associated with imprudent DH and impaired cognitive performance [59, 60] which in turn is associated with ADHD [61]. Our study showed a relation, where positive association was found between frequency of breakfast and daily vegetable consumption in both groups, while both previously mentioned parameters are negatively related with eating in front of the screen which leads to a higher attention problem score.

In summary**,** comparing the network graphs between the children with different daily MVPA levels, it can be observed that that in the group of children who do not reach WHO recommendations on MVPA, interconnections between parameters are seen more often and their influence on each other are much stronger, especially between the DH and cognitive function domains. This could mean that by modifying one variable we could expect an influence on the whole network model. Contrarily, for children who reach WHO recommendations on MVPA, the network graph is more stable, suggesting that changes in one variable will have a smaller effect on other parameters. Furthermore, central nodes of the network graphs vary between the groups, suggesting that interventions aimed towards improvement of children’s overall health might be different. In conclusion, the major findings suggest exploring the potential of interventions involving MVPA to positively impact attention issues in children. Additionally, further findings could also inform some key aspects of experimental design to e.g., control for confounding factors.

## Limitations

In the present study, participants were recruited by convenience sampling, accordingly imposing a limitation on the generalizability of the study's findings. One more of the possible limitations was that children could under- or over-report their actual DH and PA levels. The authors acknowledge this as a major challenge in all studies reliant on self-reported data collection but note that the methodological choices within the current design were the most appropriate to limit participant burden in this complex study assessing multiple outcomes. Another limitation may be that some participants from the overall study sample did not correctly understand the meaning of the question in the questionnaire due to poor ability to read or maintain focus. Children at such a young age may misunderstand the question about actual habits and did choose the answers which represent their wishful lifestyle habits. To reduce this limitation, electronic questionnaires were designed to be as short as possible and illustrated with pictures. As well, there may be limitations in cognitive test results due to not listening or ignoring instructions given by the research team. To reduce this limitation there was a trial for every test before the real attempt. In addition, a researcher or a schoolteacher was available for any questions while children filled out the questionnaires and cognitive tests. Slight deviations in the collection of anthropometric measurements by school nurses could be present, despite the provided instructions. This paper represents the baseline cross-sectional data of a longitudinal, prospective study, so any direction of association between the variables cannot yet be determined. Follow-up data will be collected in subsequent work.

### Supplementary Information

**Supplementary Material 1.**

## Data Availability

The datasets used and/or analysed during the current study are available from the corresponding author on reasonable request.

## Abbreviations

PA: Physical activity
DH: Dietary habits
MVPA: Moderate to vigorous physical activity
ADHD: Attention deficit hyperactivity disorder
PACH study: Physical activity and children overall health study
SFAC: Sport for all class
METy: Youth metabolic equivalents
BMI: Body mass index
WHO: World health organisation
EBIC: Extended Bayesian information criteria
