# Supplementary material for: Relations between the levels of moderate to vigorous physical activity, BMI, dietary habits, cognitive functions and attention problems in 8 to 9 years old pupils: network analysis (PACH Study)
Source: BMC Public Health. 2024 Feb 21;24:544. doi: 10.1186/s12889-024-18055-2 (PMC10882845; doi:10.1186/s12889-024-18055-2)
Supplement: Supplementary file 1 — Supplementary Material 1. [file 12889_2024_18055_MOESM1_ESM.docx]

**Appendix**

**Table A1.** Attention Problem Survey

| **Survey Item**  **Nr.  *Answers and scores 0=Not true 1=Somewhat or sometimes true 2=Very true or often true*** | |
| --- | --- |
| 1 | I act too young for my age |
| 2. | I fail to finish things that I start |
| 3. | I have trouble concentrating or paying attention |
| 4. | I have trouble sitting still |
| 5. | I feel confused or in a fog |
| 6. | I daydream a lot |
| 7. | I act without stopping to think |
| 8. | My schoolwork is poor |
| 9. | I am inattentive or easily distracted |


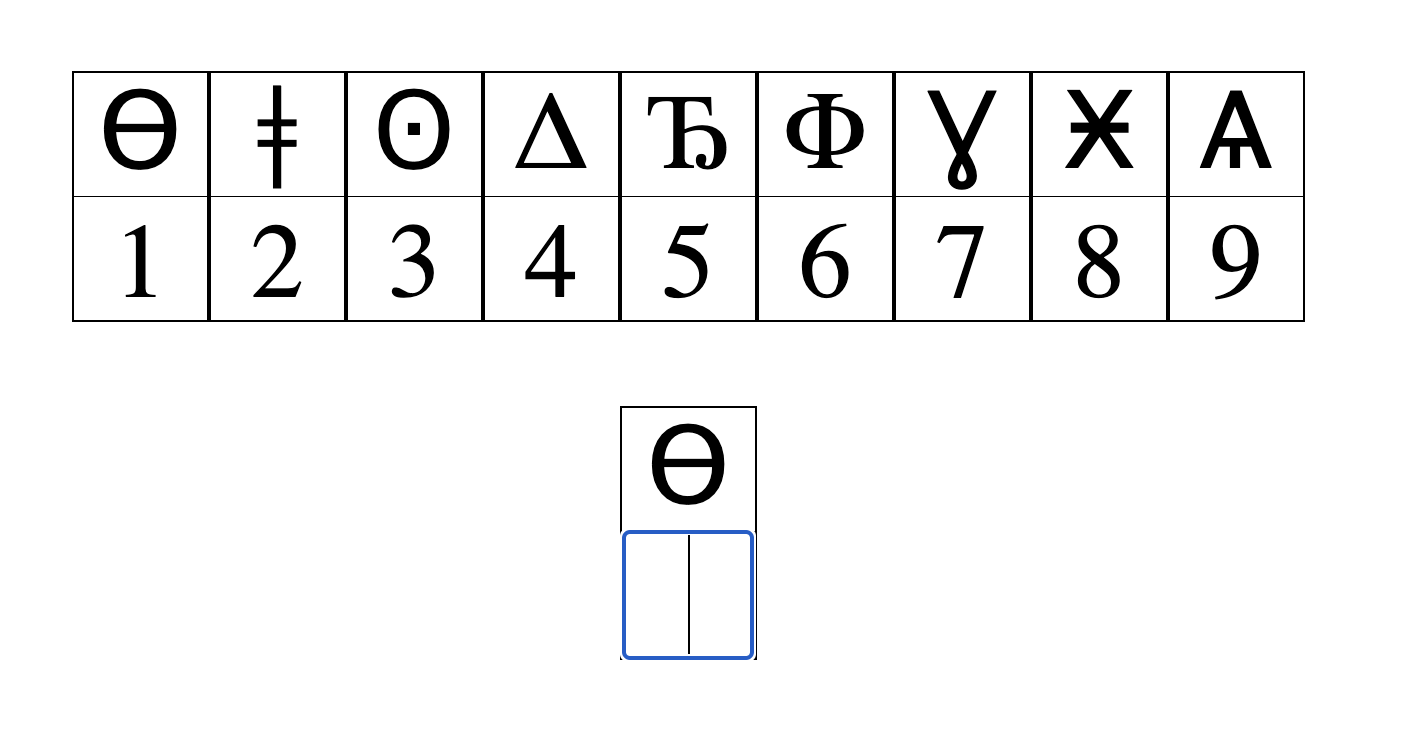


**Figure A1.** Symbol digit test.


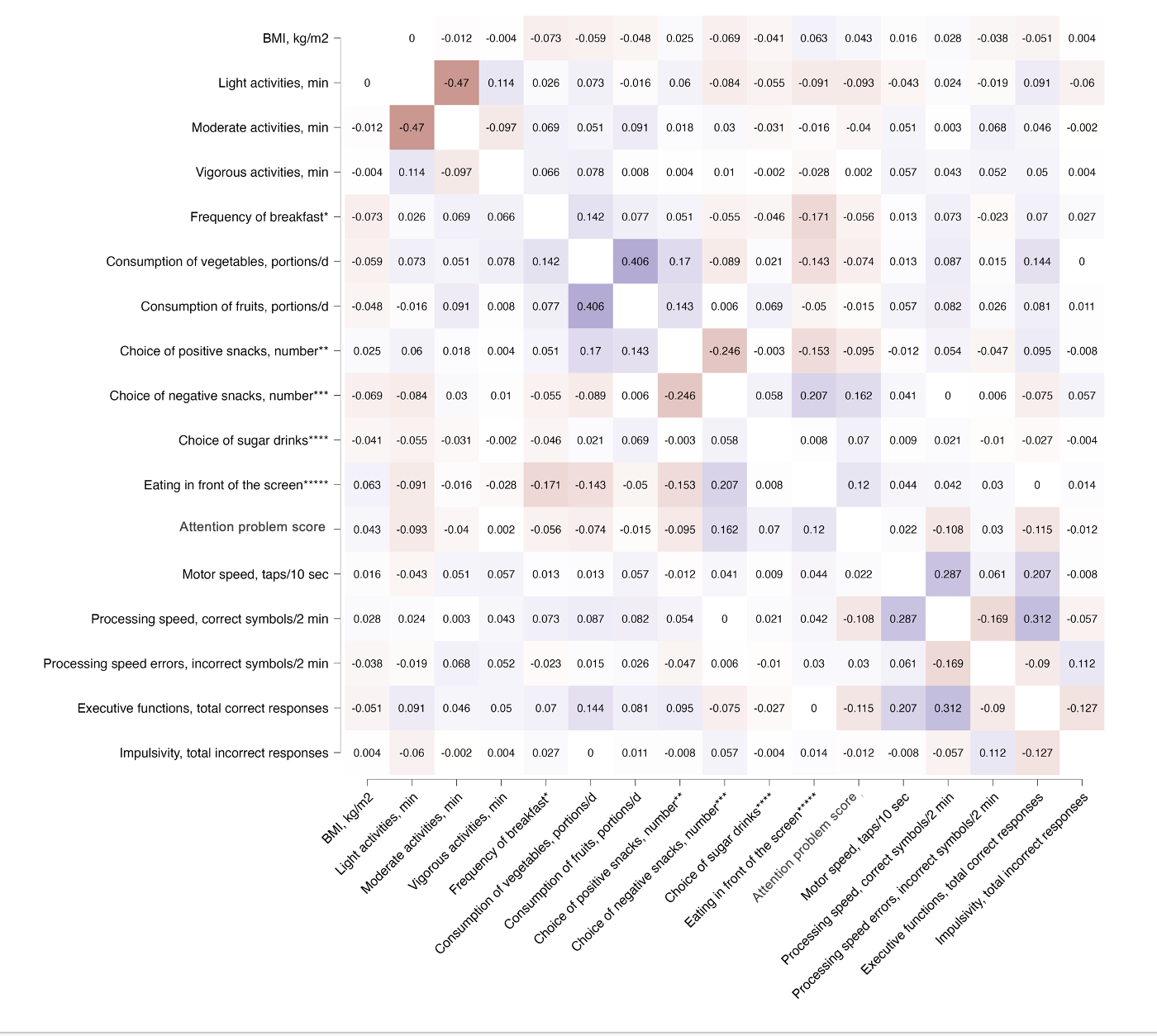


**Figure A2.** Partial correlation matrix. *p < 0.05; **p < 0.01; ***p < 0.001.

In Legend:

* 1 - never; 2 - sometimes; 3 - almost every day; 4 - every day;

** sum of nuts/dried fruits, fruits, yoghurt;

*** sum of cookies, sweet bakery, salty nuts, French fries, popcorn, chips, chocolate, chocolate sweets, ice - cream;

**** 0 - water; 1 - tea; 2 - soft drinks/sweetened juices

***** 1 - no, 2 – yes


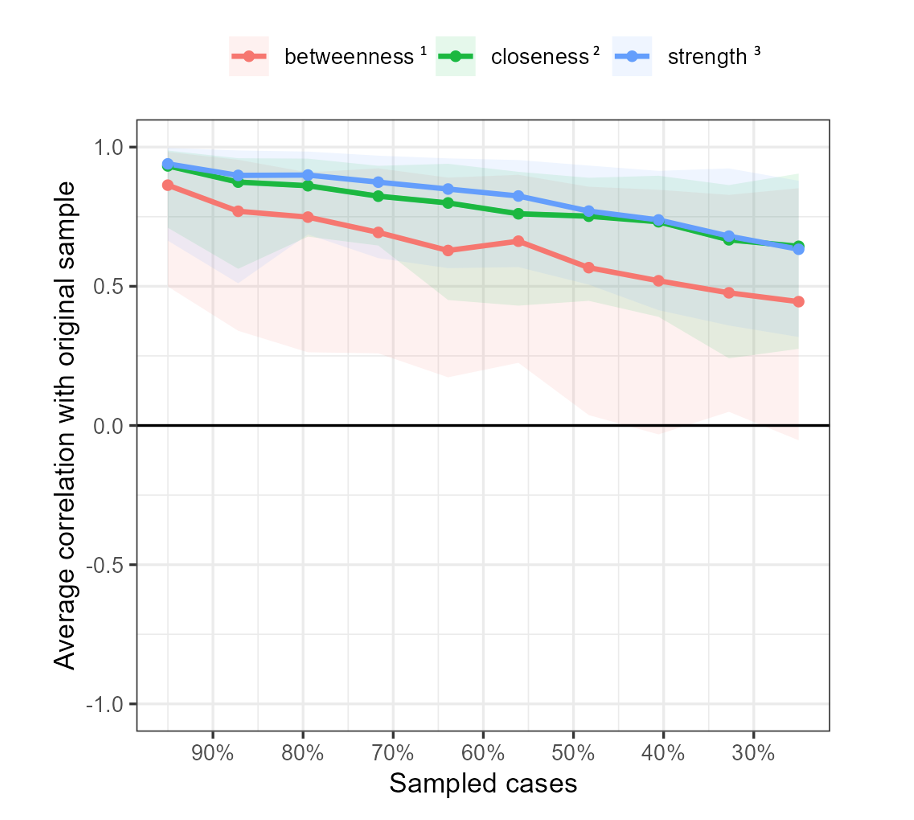


**Figure A3.** Average correlations between centrality indices of networks which are sampled with individuals dropped and the original sample. Estimates become unstable if correlation drops <0,7.

^1^ Betweenness - How well one node connects other nodes (the number of times that a node lies on the shortest path between two other nodes);

^2^ Closeness - How strongly a node is indirectly connected (average distance from the node to all other nodes in the network);

^3^ Strength - How strongly a node is directly connected (absolute sum of edge weights connected to a node).


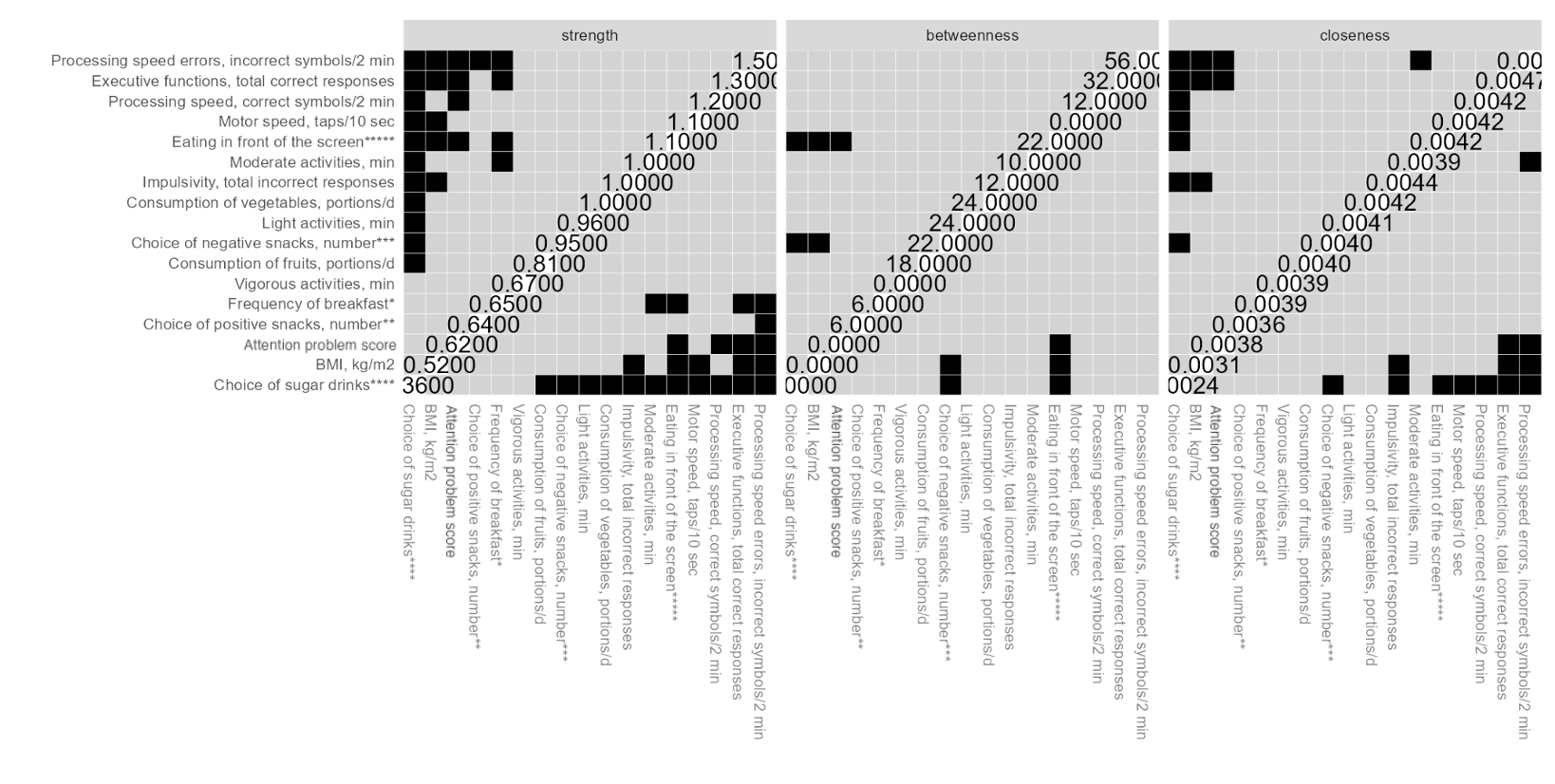


**Figure A4.** Bootstrapped difference tests (α = 0.05) between node strength, betweenness and closeness of the study measures. Grey boxes indicate nodes that do not differ significantly from one-another and black boxes represent nodes that do differ significantly from one another. White boxes in the centrality plot show the value of node strength.

In Legend:

* 1 - never; 2 - sometimes; 3 - almost every day; 4 - every day;

** sum of nuts/dried fruits, fruits, yoghurt;

*** sum of cookies, sweet bakery, salty nuts, French fries, popcorn, chips, chocolate, chocolate sweets, ice - cream;

**** 0 - water; 1 - tea; 2 - soft drinks/sweetened juices

***** 1 - no, 2 - yes
